# In vivo and qualitative studies investigating the translational potential of microneedles for use in the older population

**DOI:** 10.1007/s13346-017-0393-4

**Published:** 2017-05-15

**Authors:** Helen L. Quinn, Carmel M. Hughes, Ryan F. Donnelly

**Affiliations:** School of Pharmacy, Medical Biology Centre, Queen’s University Belfast, 97 Lisburn Road, Belfast, BT9 7BL UK

**Keywords:** Microneedles, Transdermal, Older people, Optical coherence tomography, Transepidermal water loss

## Abstract

Microneedles (MNs) are a novel transdermal drug delivery platform, rapidly progressing from a substantive evidence base, towards commercialisation. As part of this transition, it is important to consider the future use of MNs by older people in order to ensure optimal therapeutic outcomes for this unique and increasing population group. This paper, therefore, considers the use of MNs by those aged over 65 years, investigating insertion parameters in ageing skin, alongside the feasibility and acceptability of the technology. Hydrogel-forming MN arrays were applied to seven subjects aged over 65 years, with breach of the *stratum corneum* confirmed using optical coherence tomography. Insertion depths recorded in each case were similar to a comparative group, aged 20–30 years. Skin recovery was, however, demonstrated to occur at a slower rate in the older subjects, as measured using transepidermal water loss. Qualitative methods, including focus groups and semi-structured interviews, were employed to collect the views and opinions of older people and community pharmacists respectively. The overall consensus was positive, with a number of benefits to MN-mediated drug delivery identified, such as reduced dosing frequency, improved adherence and an alternative delivery route where oral or injectable medication was precluded. Concerns centred on practical issues associated with age-related functional decline, including, for example, reduced dexterity and skin changes. The presentation of this work collectively provides the first convincing report of the importance of further translational research in this area to support future MN use in older people, ensuring an age-appropriate delivery platform.

## Introduction

It is evident that the global population is ageing, with the number of people aged over 65 years increasing faster than any other age group [1]. Care of this increasing population presents a challenge, particularly given the typically high proportion of associated morbidity and mortality [2]. Medication is, therefore, a crucial intervention in the management of ageing, exemplified by the fact that more than half of those aged 65 years and over take at least three regular prescribed medicines [3]. Considering new platforms for drug delivery, microneedle (MN) technology is progressing towards relevant clinical applications, offering a promising method for transdermal drug delivery [4]. MN arrays consist of a plurality of tiny projections on the micron scale, arranged on a supporting base-plate and designed for application to the skin. The primary goal of such an approach is an increase in the number of drug candidates for transdermal delivery, made possible by circumvention of the rate-limiting barrier of the skin. MNs cause temporary disruption of the *stratum corneum*, creating microscopic aqueous pores through which drugs can diffuse to the dermal microcirculation for subsequent systemic absorption. Transdermal drug delivery has a number of pertinent advantages for the older adult, offering a viable alternative to oral delivery, with the potential to provide sustained release and minimise adverse effects [5]. Aligning these opportunities alongside the typically high medication consumption of the older population, it follows that the specific use of MNs by older people must be given due consideration to ensure the greatest patient benefit.

The performance of an MN array, as an effective drug delivery device, depends principally upon successful insertion of the needles into the skin [6]. A tangible difference in the skin occurs as a person ages, indeed, with these changes being one of the greatest visual determinants of a person’s increasing age. Increased dryness and decreased elasticity can be observed, with a number of other changes noted, including epidermal thinning and decreased dermal vascularity [7], in addition to a reduced rate of barrier repair [8]. It is, therefore, clinically reasonable to assume that the skin response to MN application in older adults may be different when compared to those younger. A recent study used impedance spectroscopy to examine skin recovery following MN treatment in older people [9], reporting that micropore closure was slower in older subjects when compared to a younger control group [9]. As yet, however, there have been no detailed studies that have investigated MN insertion and the associated parameters in older people.

When developing a new technology for drug delivery, consideration must be given to the patient who will be using it, as well as other involved parties, for example, the healthcare professionals who will be prescribing, dispensing and, in some cases, administering the device. The acceptance of a pharmaceutical product is closely linked to the success of the innovation, both in commercial terms and also therapeutically. The latter may be predicted, as patient acceptance of a device for drug delivery is typically interlinked with adherence, a vital element in ensuring optimal treatment efficacy and expected therapeutic outcomes [10]. By engaging with the patient early in the development process, potential issues can be identified, which allows these factors to be addressed and relevant adaptations to be made if necessary, ensuring that the greatest benefit can be derived from the end product. To date, studies investigating perceptions of MNs have yielded positive results, with members of the general public, children and healthcare professionals all able to identify a number of advantages to MN use, whilst also highlighting where improvements in MN patch design may be made [11–15]. There have been no studies conducted, however, that have specifically considered the views and opinions of older people, and those involved in their care, on MNs for drug delivery, despite the potential complexities of their treatment.

This study adopts a dual approach to assessing MN suitability in older people, encompassing both the performance of a MN array and the perspectives of end users and stakeholders. Optical coherence tomography (OCT) was employed as a technique to gain a full picture of MN insertion in older adults when compared to those younger, specifically considering the depth and width of the created micropore, with transepidermal water loss (TEWL) used simultaneously to map the recovery of the skin following MN removal. Qualitative methodologies were then also used to ascertain the views and opinions of those aged over 65 years (focus groups) and community pharmacists (semi-structured interviews), as a relevant healthcare professional, on the potential use of MNs for drug delivery in the older population.

## Materials and methods

### Chemicals

Gantrez® S-97, a copolymer of methyl vinyl ether and maleic acid, (PMVE/MA, molecular weight 1,500,000 Da) was a gift from Ashland, Kidderminster, UK. Poly(ethylene glycol) (PEG; molecular weight 10,000 Da) was purchased from Sigma-Aldrich, Dorset UK. All other chemicals used were of analytical reagent grade. Elastoplast® Invisible Protection plasters were obtained from Beiersdorf UK Ltd., Birmingham, UK.

### Preparation of hydrogel-forming microneedle arrays

MN arrays were prepared from aqueous blends containing 20% *w*/*w* Gantrez® S-97 and 7.5% *w*/*w* PEG 10,000. The blend (500 mg) was poured into laser-engineered silicone micromoulds to form a MN array composed of 121 (11 × 11) needles, perpendicular to the base and of conical shape, 600 μm high, on a 0.5 cm^2^ area, with a MN-free border to give a final patch area of 1.0 cm^2^. The filled moulds were centrifuged at 2205×*g* for 15 min and dried at room temperature for 48 h. MNs were then cross-linked (esterification reaction) [16] by heating at 80 °C for 24 h, and the sidewalls formed by the moulding process removed using a heated blade. Finally, each MN array was affixed to an Elastoplast® Invisible Protection plaster for application purposes.

### Participant recruitment

Ethical approval for the study was granted from the School of Pharmacy Ethics committee at Queen’s University Belfast, prior to commencement (014PMY2014, 018PMY2015). Written informed consent was then obtained from subjects, before enrolment in any part of the study. All participants in the MN insertion experiment were either aged over 65 years, forming the group under investigation, or aged 20–30 years, acting as a control group. All had no pre-existing skin conditions or allergy to plasters. Regarding participation in the focus groups, apart from an age of 65 years or older, there were no further inclusion or exclusion criteria. Older participants for both parts of the study were approached and invited to participate through a local charity engaged in service provision for the over 65 years old age group. The participants aged 20–30 years old were all employed at the School of Pharmacy, Queen’s University Belfast, recruited by means of an email circular. The only criterion for community pharmacist interviewees was that they had to be practicing in Northern Ireland at the time of the interaction.

### Insertion study procedure

The study was conducted at a controlled room temperature of 20 °C within the School of Pharmacy, Queen’s University Belfast. As the same environment was used throughout, room humidity was not measured, which was deemed to be consistent for each study participant. Subjects rested within the room with their ventral forearms uncovered for at least 15 min prior to the start of the experiment to acclimatise. Two areas on the participant’s ventral forearm were marked using a washable pen and numbered 1 and 2. A baseline TEWL reading was taken from the first marked location on the arm, using a VapoMeter® (Delfin Technologies Ltd., Kuopio, Finland), a closed-chamber, portable system which operates via a sensitive humidity sensor. The sensor monitors the increase of relative humidity inside the chamber during the measurement, allowing the evaporation rate value to be automatically calculated from the relative increase. The TEWL values were obtained by placing the meter on the relevant area on the forearm of the participant, with the probe head vertical and perpendicular to the skin, ensuring that the area of skin to be analysed was sealed by the adapter. Skin contact was maintained for approximately 10 s before generation of an evaporation rate value. The researcher then applied an MN patch to this area, using thumb pressure, in the direction of the skin, held for 30 s and timed using a stopwatch. Once inserted, the MN array was visualised in situ using the handheld probe of the VivoSight® topical OCT system (Michelson Diagnostics Ltd., Kent, UK). The swept-source Fourier domain OCT system has a laser centre wavelength of 1305 ± 15 nm, facilitating real-time high-resolution imaging of the upper skin layers (depth ∼ 2 mm, <7.5 μm lateral and <10 μm vertical resolution). The resultant 2D images from OCT scans were analysed using the imaging software ImageJ® (National Institute of Health, MD, USA). The scale of the image files obtained was 1.0 pixel = 4.2 μm, thus allowing accurate measurements of the depth of MN penetration and the width of pore created in the skin. For each subject, OCT data were presented as mean (±S.D.) of 12 replicate measurements of individual MN insertion depth and the diameter of the corresponding micropore, where the measured MNs were selected at random from the 121 penetrating needles in each array. The MN patch was removed from the skin following OCT use, and a TEWL measurement of the area was taken immediately. This area of skin was then left untouched for 30 min before a final TEWL measurement was recorded.

The second MN application was performed on the location labelled number 2 on the ventral forearm; this time applied by the volunteer themselves. As before, a baseline TEWL measurement was taken from this location prior to application. The subject was then asked to apply the MN patch by replicating what the researcher had done previously. In this way, no formal counselling was provided and the participant had to rely on experience from the former application. As described previously, OCT scans were taken of the inserted MN array, prior to its removal. TEWL measurements were taken immediately post removal and after 30 min.

### Questionnaire

A short, structured questionnaire, consisting of eight fixed questions, was devised to collect demographic data from the insertion study participants, as well as their views and opinions of the application process they had just experienced, with their answers based on a Likert scale. Questionnaires were designed in consultation with MN and pharmacy practice researchers and piloted with staff and post-graduate students in the School of Pharmacy to ensure understanding of questions and ease of response.

### Focus groups

**Fig. 1:**
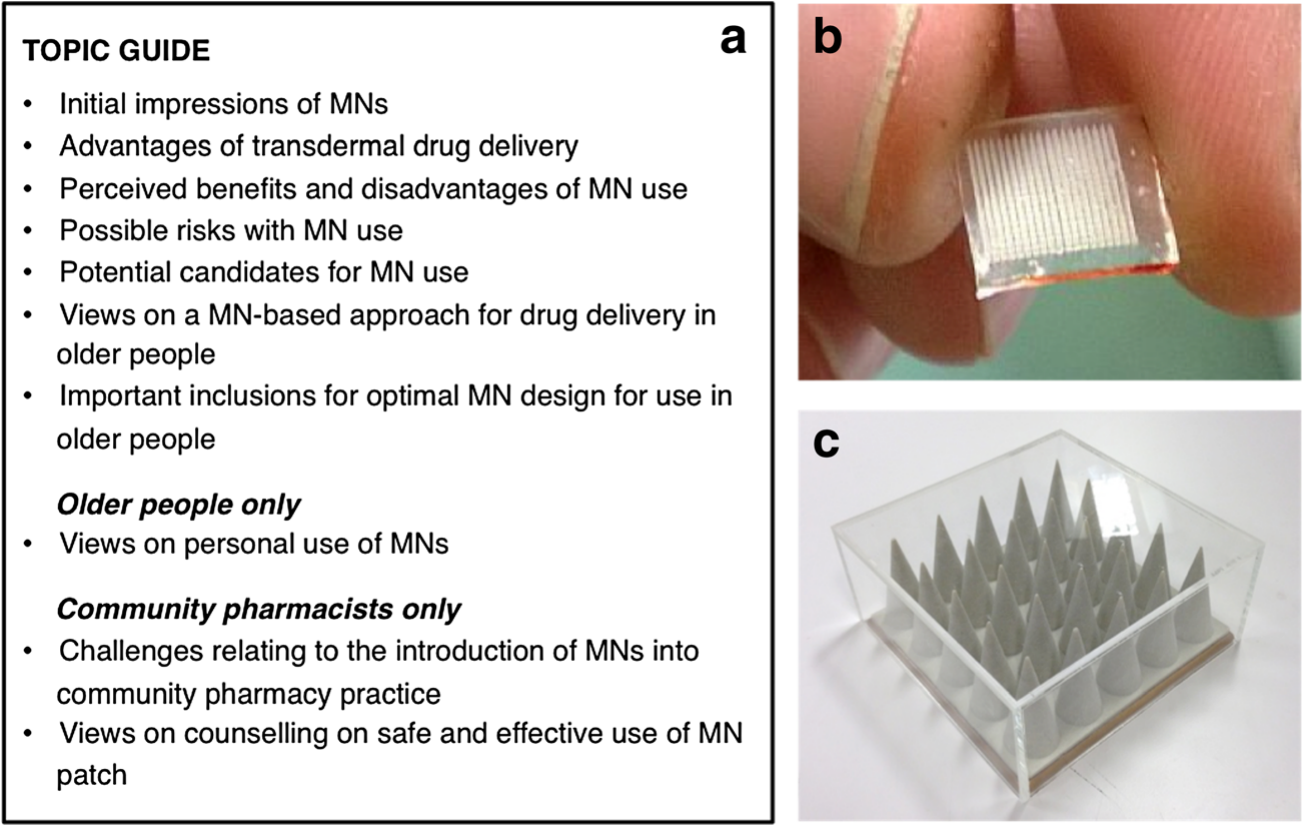
Topic guide for focus groups and semi-structured interviews (**a**); 1 cm^2^ polymeric MN array (**b**); and oversized MN array enclosed within a Perspex box (**c**), both used for demonstration purposes

Focus groups were employed to assess the perspectives of older people on the use of MN technology. Such a methodology has been deemed particularly useful when knowledge on the issue in question is lacking, which, given the novelty of this topic, was the context for this study [17]. The group interaction promotes discussion, thus leading to a greater appreciation of older people’s views than one-to-one questioning [18]. A topic guide for focus group discussion was developed according to previous studies investigating MNs using qualitative methodologies [11–13, 15], and then piloted to refine content and ensure validity. Each focus group began with simple introductory questions relating to medication use and common issues encountered, before progressing to the concept of MNs and discussion points on the use of such a technology by older people, as summarised in Fig. 1a. To facilitate discussion, an introduction to MNs was provided using a short presentation, explaining what they were and their potential role in drug delivery. Sample MN arrays, produced for experimental purposes (Fig. 1b), were distributed for inspection, as well as an oversized prototype MN array contained within a sealed Perspex box (Fig. 1c). Each focus group ended with a short summary of the discussion and the opportunity to ask any further questions.

### Community pharmacist interviews

Semi-structured interviews were used to investigate the views and opinions of community pharmacists on the use of MNs by older people. It was anticipated that pharmacists would have knowledge of emerging drug delivery technologies, in addition to experience of medication management in older adults; hence, interviews were viewed as suitable to focus on the topic and achieve an appropriate level of detail. The interviews were designed to follow a similar semi-structured topic guide to that used for focus groups, developed and piloted as previously described. As an introduction, the interview began by establishing if the pharmacist had any prior knowledge of MNs. Irrespective of the answer, an explanation of MNs was then provided, using a short presentation to explain what MNs were and how they worked, with an appropriate level of detail for a healthcare professional. The main interview questions then related to the use of MN technology in older people and the challenges and opportunities relating to the introduction of such a technology into community pharmacy practice, as summarised in Fig. 1a.

### Qualitative data handling and analysis

All focus groups and interviews were audio-recorded using a digital voice recorder (Olympus WS-321M, Olympus Corporation, Tokyo, Japan). Recruitment and data collection ceased when data saturation was deemed to have occurred, noted as the appearance of no new themes emerging with subsequent focus groups or interviews. Audio recordings were transcribed verbatim, all identifiers removed and codes assigned to participants (to ensure anonymity) before being imported to NVivo® (QSR International Pty Ltd., Doncaster, Australia) for thematic analysis. Broad themes were identified and analysed by constant comparison between and within transcripts. By including both community pharmacists and older people, the research question was explored from various perspectives, a concept known as data triangulation, an approach credited with improving the validity of qualitative study findings [19]. Common themes were then grouped together into subthemes, and following further refinement, core themes, identified to accurately represent the entire data set. Consensus on the emergent themes was reached by discussion among all three researchers (HQ, CH, RD).

### Statistical analysis

Where appropriate, data was analysed using a *t* test, Mann-Whitney *U* test or a one-way analysis of variance (ANOVA), with post-hoc comparison performed using Tukey’s HSD test. In all cases, *p* < 0.05 denoted significance.

## Results

### Insertion study subjects

A total of 15 people were recruited to the insertion study. Seven participants were aged over 65 years (four male), with a maximum age of 82 years, and eight were aged between 20 and 30 years (four male). MN application was well tolerated by all subjects, with no local reactions or adverse effects observed over the course of the study.

### Optical coherence tomography

**Fig. 2:**
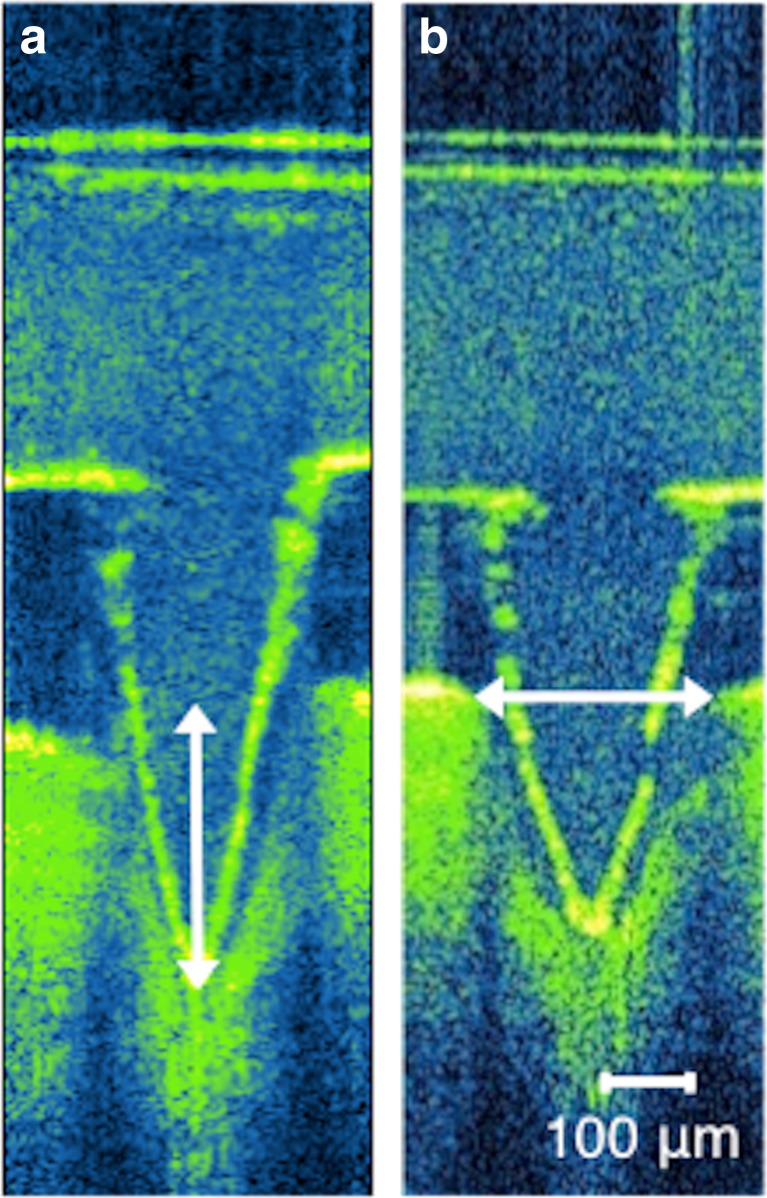
Representative OCT image of a MN, displaying the measurement of the insertion depth (**a**) and pore width (**b**) upon application

The mean insertion depths and pore widths upon MN application were measured as shown in Fig. 2, and the resultant findings are presented in Table 1. Comparing researcher and self-application within each age group, in the 20–30 year olds, there was no difference in the resulting insertion depths (*p* = 0.18), whereas in the ≥65 year olds, researcher application resulted in significantly lower insertion depths than when self-applied (*p* = 0.016). Indeed, the insertion depths resulting from researcher application of MN patches in the ≥65 years age group were found to be significantly lower than both researcher and self-application in the 20–30 year olds (*p* = 0.0014). However, there was shown to be no difference between the MN application in the 20–30 year olds and the self-application in the ≥65 year olds. There was no significant difference in the pore widths created by MN insertion in any of the application types or age groups (*p* = 0.30).

**Table 1:** Insertion depths and pore widths measured from polymeric MN application, by the researcher and by the subject themselves (mean ± S.D., *n* ≥ 7)

Age (years)	Applicator	Insertion depth (μm)	Pore width (μm)
20–30	Researcher	356 ± 21.6	279 ± 24.0
	Self	371 ± 16.1	283 ± 13.8
65	Researcher	321 ± 14. 8	292 ± 11.7
	Self	347 ± 30.3	294 ± 19.0

### Transepidermal water loss

**Fig. 3:**
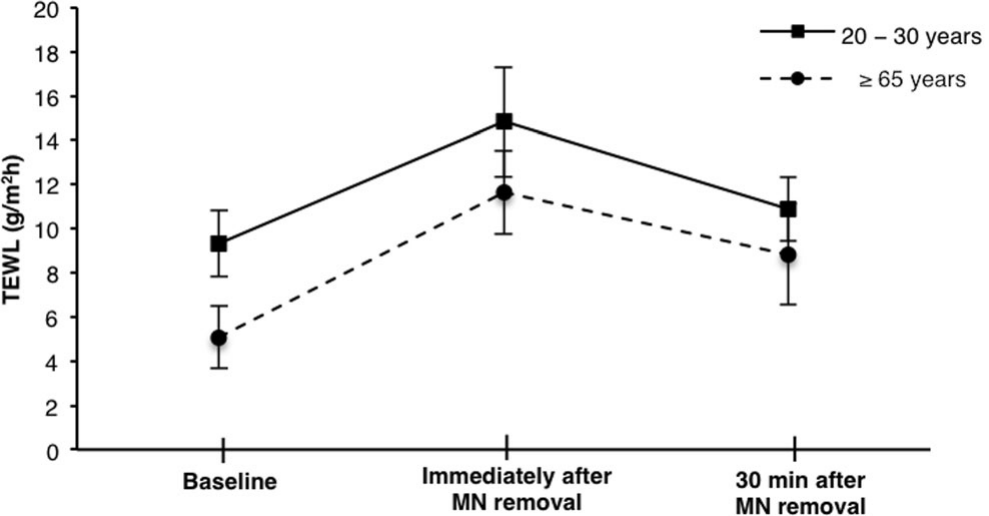
Change in TEWL from baseline following MN application and removal up to 30 min (mean ± S.D., *n* ≥ 7)

The initial baseline TEWL differed significantly between the two age groups (*p* < 0.0001), with a mean value of 4.94 g/m^2^ h measured in the ≥65 year olds, compared to 9.31 g/m^2^ h in the 20–30 year olds. Upon MN application and subsequent removal, there was a significant increase in TEWL in both age groups, which decreased after 30 min but remained significantly higher than baseline (Fig. 3). In the 20–30 year olds, the percentage difference from baseline after 30 min was only 17.59% whereas in the older age group, the percentage change remained high, at 71.61% greater than the original value.

### Post-application questionnaire

All participants were positive about MN technology, with 100% of participants willing to use them again in the future. Similarly, all were satisfied or very satisfied with the ease and comfort of MN application and confident or very confident in applying the MN patch to themselves.

### Focus group and interview participants

A total of five focus groups were conducted, with 7–10 people in each, providing an overall total of 41 participants, ranging in age from 65 to 86 years. Regarding interviews, a total of 12 community pharmacists took part, with the mean number of years post registration as a pharmacist being 12.2. Focus groups lasted, on average, 67 min, whilst pharmacist interviews lasted approximately 22 min. All participants were assigned a unique identifier code such as ‘FG1_M_67’, which indicated that this was a member of focus group 1, male, aged 67 years. If there was more than one person of the same age and sex in the same group, A, B and C were used to further differentiate the participant. For the interviews, codes such as ‘CP_3’ were employed, which referred to community pharmacist number 3, numbered according to the sequence of the interviews conducted.

### Main themes

**Fig. 4:**
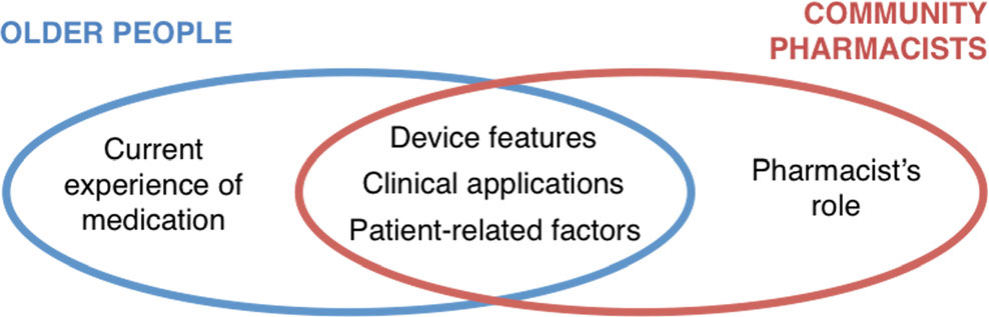
Schematic illustration of themes identified from focus groups with older people and semi-structured interviews with community pharmacists

Following detailed thematic analysis, five themes were identified, as illustrated in Fig. 4, three of which were common across the focus groups and interviews: device features, clinical applications and patient-related factors. One theme resulted from focus group discussions only, namely, current experiences of medication, and one further theme from the interviews only, that is, the pharmacist’s role.

#### Device features

The features of a MN product were extensively discussed as older people contemplated their ability to handle such a patch. The size and colour were particular areas of focus, with the acknowledgment that both would have a large impact on an older person’s handling of a MN patch. The packaging of the device was also considered, as older people explained the frequent difficulties encountered in removing medication from its packet. Participants were positive about the possible inclusion of a colour change upon application of pressure and insertion, remarking that “it would give older people confidence, knowing that they were taking their medication right” (CP_9).

#### Clinical applications

A number of drugs were suggested for delivery via MNs, some chosen according to the circumstances of the patient and others according to the properties of the drug. For example, donepezil for treatment of dementia in Alzheimer’s disease was named by a number of participants, with the rationale that patients prescribed this drug often demonstrated swallowing and/or adherence issues. The other type of drug most frequently suggested was that which provoked gastrointestinal adverse effects, e.g. non-steroidal anti-inflammatory drugs (NSAIDs), as, if using a MN patch, “you wouldn’t have the side effects of it upsetting your stomach or your bowel because it’s not going that way” (FG5_F_85). The community pharmacist interviewees expressed reluctance towards the incorporation of drugs that would require regular monitoring or frequent dosage alterations, with a preference noted for long-term medication on which the patient was stable. Participants also recognised the opportunity for sustained delivery of drugs using MNs, identifying this as a further positive feature which may reduce adverse effects and improve adherence, “instead of having to take a statin [cholesterol-lowering drug] every day, you’ll be able to put a patch on and it will last for a week” (FG1_M_70A).

#### Patient-related factors

The utility of MNs as an alternative delivery route where patient circumstances may preclude the use of other common routes for medication administration was highlighted as being particularly beneficial in the older population, and indeed, this is where a number of participants saw the principal advantage of MN use in this group. Difficulty in administration of injections, poor or no swallow, alongside refusal to take oral preparations in cognitively impaired patients were the main problems cited, of which some of the participants had personal or professional experience.

The majority of participants believed older people would be able to apply a MN patch effectively, comparing it to application of a sticking plaster or a conventional transdermal patch. Loss of dexterity and vision were cited as the most likely barriers to the practical use of a MN device, although if designed appropriately, these were not believed to be limiting factors. Another issue discussed was the changes observed in the skin upon ageing, with apprehension expressed as to whether these differences could have an impact on effective MN usage. Similarly, concern was expressed whether older people would be more susceptible to damage or irritation, due to changes in hydration and the increased fragility of ageing skin.

Considering the acceptability of a MN product, older people rejected the stereotype often associated with their age group, stating that, “it’s not really an assumption to make, in that because somebody is older, that they won’t be open to a new idea” (FG3_F_74). Although it was acknowledged that there was likely to be some reluctance about a change from an accepted conventional delivery form, such as a tablet, to a MN patch, appropriate education and professional advice were described as sufficient to overcome any concerns.

#### Current experiences of medication

Older people reported a complex situation when asked about their current experiences of taking medication. The combination of an uncertain relationship with the prescriber, limited information regarding their medication and practical issues regarding medicine management and administration frequently led to non-ideal medication use. There was little experience of using transdermal patches by the older people involved, although participants were aware there was only a limited number of transdermal patches currently available for drug delivery.

#### Pharmacist’s role

The pharmacists identified their primary role to be in counselling patients on MN use following supply, deemed to be particularly important, considering the novelty of MNs. Pharmacists expressed confidence regarding counselling on an MN patch, suggesting a number of supporting materials to assist with the counselling process, including a placebo patch, training video and diagrams, in addition to the patient information leaflet. The main barrier to implementation of the counselling strategies proposed was having the time that would be required to ensure understanding, rather than the age of the patient per se.

## Discussion

As a novel drug delivery technology, the future use of MNs across the entire population must be carefully considered to ensure the greatest impact in terms of both patient and commercial outcomes. This is of particular relevance for the older population, a heterogeneous group who may demonstrate different needs to younger adults, in terms of their pharmaceutical care [20]. The present study was, therefore, conducted in order to assess the applicability of MNs for drug delivery specifically in those aged over 65 years.

In the first instance, the practical application of MNs to ageing skin was considered, focusing on insertion of the needles and the subsequent recovery of the skin following MN removal. MN arrays of a previously optimised and validated design, in terms of material, geometry and needle dimensions, were employed [21], with the variable being the age of the subject. Further to this, in order to investigate the ability of older people to self-apply the MN arrays, one application was performed by the subject to him or herself, in addition to the application carried out by the researcher. Overall, from the two applications, the mean insertion depths obtained ranged from 60.6% of the MN height penetrating in the 20–30 year olds to 55.7% in those aged over 65 years, which is in line with recent studies investigating the insertion of this design of polymeric MNs [22, 23]. This observation demonstrates the relative similarity of MN insertion in the two age groups. A significant difference was, however, noted between the insertion depths of the MNs in the arrays applied by the researcher to the older subjects, in comparison to both applications in the younger group. When self-applied by the older subjects, this difference was not observed, with the mean insertion depth comparable to the 20–30 year olds. This implies that the lower insertion depth observed initially may be due, at least in part, to the application process, as conducted by the researcher, who may have exercised excessive caution in terms of the pressure applied to the array due to the age of the subject. The other theory for the lower insertion depth, as previously hypothesised in the literature, is that a greater force may be required for MN insertion in ageing skin [24]. Nevertheless, in all cases, the MNs efficiently traversed the *stratum corneum* and, with a maximum range between insertion depths of only 49.4 μm, any consequences for drug delivery are likely to be arbitrary [25, 26]. Indeed, with hydrogel-forming MNs, once the array begins to absorb interstitial fluid from the skin and swell, little difference in penetration depth is observed from that point on, minimising any effect from initial differences [27]. The other parameter measured upon MN insertion was the width of the micropore formed in the skin. Typically, due to the conical shape of the needles, the greater the insertion depth, the greater the width of the pore it creates in the skin. Although the mean insertion depth for those aged over 65 years was lower than the 20–30 years old, the corresponding mean pore widths were higher, albeit not significantly. One possible theory for this disparity is due to the decreased elasticity of ageing skin, which may be contributing to a decreased enveloping of the needles when they puncture the skin surface, hence, creating a larger pore diameter around each MN [25]. For MNs which remain in place for the duration of drug delivery, such as the hydrogel-forming platform used in this study, the size of the pore is of little consequence for drug diffusion, as the permeant moves through the MN matrix itself; however, such a finding may have implications for the recovery of the skin.

It is naturally of great importance that MNs do not cause lasting damage to the skin and the integrity of the barrier can be restored upon MN removal. TEWL was, therefore, used as a means of measuring skin recovery in the present study. The baseline TEWL recorded was significantly lower in the older sample group than in the younger age group, which, despite appearing counter-intuitive, is in line with previous findings of TEWL in the older population [28]. The general pattern of the changes in TEWL following MN application and removal was as expected, with a significant increase noted immediately upon MN removal and a subsequent decrease after 30 min [29]. Importantly, the trend observed was similar between the two age groups. There was a greater percentage increase in TEWL immediately following MN removal in the older group (143.1% compared to 59.47%), which remained notably above baseline after 30 min. This may indicate that ageing skin recovers at a slower rate than that of younger counterparts, which could be linked to the decreased elasticity and declined rate of barrier repair, common in ageing skin [30]. Further measurement of TEWL beyond 30 min would be required to confirm this theory, however. Importantly though, there was a 28.0% decrease in the TEWL of the over 65 year olds over the 30 min period following MN removal, demonstrating that the skin is capable of recovering within a reasonable time frame.

The favourable attitudes towards MNs revealed by the qualitative investigations echo the results of prior studies on the acceptability of a MN platform for drug delivery [11, 15, 31]. Older people highlighted the perceived benefits of MNs as an alternative route for drug delivery, in contrast to the issues often experienced with both oral preparations and injectables. For example, swallowing difficulties are known to be common in the older population, associated with increasing age and the presence of certain conditions such as Parkinson’s disease and dementia [32]. Delivery via injection may also be more problematic in older people, due to limited venous access as a result of low body weight and decreased muscle mass, often associated with frailty [33]. Transdermal therapy was indicated as being extremely useful in these circumstances, permitting medication administration with ease and maximising patient convenience. Indeed, the participants in the present study noted a potential improvement in adherence with MN use, an advantage not previously identified. There are existing reports of improved adherence in the older population due to transdermal drug delivery, with specific examples relating to the treatment of Alzheimer’s disease [34, 35]. Such improvements in adherence may be due in part to the ability of a caregiver to effectively facilitate application of a transdermal patch, in comparison to the difficulties faced in administration of an oral preparation to a patient or family member, who may be exhibiting severely reduced mental and or physical capacity. The use of MNs in this context could, therefore, offer great patient benefit.

A notable advantage of a MN system is the option of self-administration, particularly when compared to hypodermic injection, which typically requires a healthcare professional for administration. It has been previously demonstrated that hydrogel-forming MNs can be effectively self-inserted by a group of undergraduate pharmacy students, with the assistance of pharmacist counselling and a patient information leaflet [23]. Further evidence would be desirable to extend this principle to older people, who often exhibit a decline in functional capacity, including decrements in cognitive, visual or motoric capabilities [36]. Considering practical handling, the decrease in motor function may manifest as difficulties in manipulation and handling of medication, due to slower, less coordinated and less controlled performances in tasks such as this which require a degree of hand dexterity [37]. Encouragingly, however, all participants in the present study were able to apply a MN patch to themselves, and stated that they were confident or very confident in doing so. The qualitative study also revealed similar confidence with respect to potential application of a MN patch, providing that size and colour were considered. Distinct from the MN array but still vital for usability purposes is the packaging of the final product. This was highlighted in both aspects of the study, with the topic extensively discussed in the qualitative interactions and assistance required in some cases during the insertion experiments to remove the backing from the adhesive plaster before MN application. The issues older people have in opening and handling medication are well known, although they are still not always recognised in product design [38].

In order to facilitate the safe and effective use of a MN product by community-dwelling older people, engagement by the pharmacy profession would be required, due to their involvement in patient counselling, particularly critical with a new drug or device [39]. The community pharmacists interviewed here clearly identified their own role in the counselling and education of the patient on the use of MNs, expressing their belief that, following their intervention, older people would be able to use MNs safely and effectively. To ensure optimal therapeutic outcomes, there is evidence to suggest that patients’ capacity to manage new medication delivery systems should be tested with simulated devices prior to home use [40, 41], reinforcing the need for a placebo MN patch for training purposes, as recommended by the pharmacists here.

Some unique questions relating to MN use were raised, specific to this age group, reiterating the value of stakeholder engagement early in the development process. Pharmacists and older people expressed concern as to whether MNs could be rendered ineffective, due to the age-related changes observed in skin or whether adaptations would need to be made to the MN dimensions for use in older people. Comparing it to conventional transdermal drug delivery using patches, there are currently no known differences observed in practice between passive absorption in young and older (over 65 years) individuals [5]. As an example, when treating those aged over 75 years compared to those aged 50–60 years, no dosage alteration of buprenorphine patches was necessary [42]. The use of MNs themselves in older people, however, as this study has highlighted, warrants further investigation. Encouragingly, to date, no unusual skin responses to MN insertion have been recorded in those over a certain age [9], despite the overt changes in skin anatomy.

There are a number of factors that should be noted, when interpreting the results and considering the further development of this work. Firstly, it must be highlighted that this study considered a small number of subjects, so any differences observed in MN insertion and skin recovery would need to be validated in larger populations. A longer follow-up period after MN application, alongside a longer insertion time where the MNs remained in place for up to 24 h, would also warrant further investigation. Considering the qualitative results, given their nature, it must be acknowledged that the data cannot be broadly generalised, although the findings may be transferable. Further to this, an explanation of MNs was given at the beginning of every interaction due to the novelty of the topic and, although strictly factual in nature, this information may have affected the responses provided.

## Conclusion

In the present study, MN insertion in ageing skin and the subsequent recovery of the skin were evaluated, highlighting successful breach of the *stratum corneum* upon application in older subjects and a degree of skin recovery within 30 min. The views and opinions of older people and community pharmacists regarding MN use were collected, providing key insights into the perspectives of those who would be involved in the end use of the technology. The likely acceptability of the technology and the engagement of community pharmacists were encouraging findings from these qualitative investigations. The presentation of this evidence collectively provides a strong basis for further research in this area and future clinical studies in this key target population.
